# Textured Building Façades: Utilizing Morphological Adaptations Found in Nature for Evaporative Cooling

**DOI:** 10.3390/biomimetics6020024

**Published:** 2021-03-29

**Authors:** Megan Peeks, Lidia Badarnah

**Affiliations:** The Department of Architecture and the Built Environment, Faculty of Environment and Technology, University of the West of England, Bristol BS16 1QY, UK; lidia.badarnah@uwe.ac.uk

**Keywords:** biomimicry, biomimetics, evaporative cooling, thermoregulation, façade panels, morphological adaptation, architecture, buildings, design

## Abstract

The overheating of buildings and their need for mechanical cooling is a growing issue as a result of climate change. The main aim of this paper is to examine the impact of surface texture on heat loss capabilities of concrete panels through evaporative cooling. Organisms maintain their body temperature in very narrow ranges in order to survive, where they employ morphological and behavioral means to complement physiological strategies for adaptation. This research follows a biomimetic approach to develop a design solution. The skin morphology of elephants was identified as a successful example that utilizes evaporative cooling and has, therefore, informed the realization of a textured façade panel. A systematic process has been undertaken to examine the impact of different variables on the cooling ability of the panels, bringing in new morphological considerations for surface texture. The results showed that the morphological variables of assembly and depth of texture have impact on heat loss, and the impact of surface area to volume (SA:V) ratios on heat loss capabilities varies for different surface roughness. This study demonstrates the potential exploitation of morphological adaptation to buildings, that could contribute to them cooling passively and reduce the need for expensive and energy consuming mechanical systems. Furthermore, it suggests areas for further investigation and opens new avenues for novel thermal solutions inspired by nature for the built environment.

## 1. Introduction

The overheating of buildings and the need for mechanical cooling is a growing issue due to climate change. It is noted that the building envelope mediates between the internal and external environment, where heat can be transferred between buildings and their environment by conduction, convection, radiation, and evaporation. Organisms maintain their body temperature in very narrow ranges in order to survive. They employ morphological and behavioral means to complement physiological strategies for adaptation. Certain skin morphologies in nature enhance thermal regulation capabilities, such as wrinkles on elephants’ skin enhance cooling via evaporation [1]. The exploitation of morphological features on building skin presents an opportunity to complement the heat regulation of buildings passively. This has been demonstrated in existing studies into the manipulation of geometry to increase a building envelope’s thermal performance by the creation of microclimates using cavities [2]. Additionally, utilizing the effects of rainwater runoff on façades has been identified to have a cooling effect [3] and heightening this through the use of textured façade panels has been suggested as an area of development [4] but has not yet been examined. However, there are many studies into the passive use of evaporative cooling. For instance, some studies utilize different material properties to maximize the effects of evaporative cooling, such as, Hydroceramic by Rathee et al. [5] which adopts the use of hydrogel to store water and Breathing Skin by Castro et al. [6] that uses Sodium Polyacrylate to store water. The development of a self-shading ceramic brick by Laver et al. [7] also demonstrates the advantages of evaporative cooling, however, this system uses irrigation to make the façade wet, rather than fully utilizing passive methods. Additionally, Rael and San Fratello [8] have developed the Cool Brick, a 3D printed porous brick that allows façades to store water and cool internal environments through the use of evaporative cooling. The shape of the Cool Brick starts to develop the texture of the façade to allow the bricks to stack and to create self-shading, but the focus of the study is on the porosity of the brick and fabrication techniques.

Therefore, from examining existing products and studies, it has been highlighted that there is a gap in existing research concerning the performance of facades by utilizing morphological features such as surface texture for evaporative cooling. In response, this design research work studies evaporative cooling in nature and identifies relevant skin morphologies for application to the built environment, in particular façade panels. Following a biomimetic design approach, this study aims to explore how texture employed on the surface of concrete panels can facilitate evaporative cooling in warm temperate environments. As a complementary passive technique, this is a potentially simple, affordable, and efficient way that could contribute to the cooling of buildings and reduce the need for expensive and energy consuming mechanical cooling systems.

## 2. Background

Background research into façade development has been undertaken followed by exploring examples of evaporative cooling found in nature.

### 2.1. Facades

The building envelope has evolved in time from being made of massive elements to becoming layered based [9], facilitating the advancement of multifunctional façades. This was illustrated by the development of the polyvalent wall (a wall for all seasons) by Davies [10] for the Lloyd’s Building, London. This demonstrates that although building façades typically act as barriers, the building envelope can mediate between the internal and external environment and provide an opportunity to contribute to the wider performance of the building, for instance, thermoregulation.

Utilizing this opportunity has also been suggested within existing literature, for example, Grobman and Elimelech [2] research into geometry manipulations to increase a building envelope’s thermal performance. They achieve this by creating a microclimate through manipulating cavity geometries. From simulating airflow across the cavities in the façade they conclude that the microclimates created can help to act as thermal insulation for buildings, reducing the need for thermal insulating materials. Furthermore, rainwater runoff on façades has been previously identified to have a cooling effect [3] and enhancing this using textured façade panels has been suggested as an area of development [4] but has not yet been explored.

These examples validate further research into the use of building façades for thermal regulation as they illustrate the possibilities for increased functionality of façades and identify gaps within existing research. It is also noted that the use of adaptive building envelopes to respond to changes in climate is relatively new within architecture, but within nature, thermoregulation is commonplace [11]. Therefore, learning from nature and taking a biomimetic approach for this research is considered appropriate.

### 2.2. Evaporative Cooling

Numerous examples of dissipating heat via evaporation can be identified in nature, such as sweating, panting and gular fluttering [12]. Evaporative cooling works by utilizing water as a heat sink, as energy is used to turn water into a gas (latent heat transfer), resulting in decreasing temperatures [13]. Table 1 presents examples of mechanisms to enhance evaporative cooling in nature.

### 2.3. Elephant Skin

Elephants are terrestrial mammals with a large volume to surface area ratio, inhabiting environments with temperatures up to 50 °C, hence they have a significant heat transfer challenge [16,17]. They have developed numerous mechanisms to deal with overheating, including ear flapping, water spraying, body temperature fluctuations, low density hair, and skin wrinkles, to name a few [16,18]. In this study we selected the texture of an elephant’s skin, as it is a morphological adaptation that holds water, allowing evaporation, which can be potentially applied to building façades.

The network of wrinkles on the surface of an elephant’s skin enhances their thermoregulation by retaining water in the crevasses across the skin allowing for 5–10 times the retention of water than a flat surface, supporting thermoregulation through evaporative cooling for a longer time [19]. Wrinkles also self-shade and create convective currents that augment cooling [20]. As elephants do not have sweat glands, they have to wet themselves by spraying themselves or bathing in water, this can be translated into utilizing rainfall and releasing it onto façades when needed.

## 3. Materials and Methods

**Figure 1:**
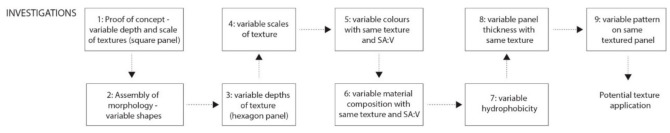
Investigations’ process. This diagram presents the process and sequence of investigations and their relevant examined variables.

A biomimetic problem-based approach has been taken for this study, starting with the problem of overheating of buildings, and using nature to inspire and develop the solution [21]. This approach has been taken so that a design solution can be meaningfully developed, by exploring evidenced solutions found in nature. Additionally, this study is based on research through design, by making and testing a series of iterations that inform consequent iterations, where the acquisition of knowledge and decision making are through the design process [22]. As such, a systematic and iterative [23] development process has been undertaken to investigate the efficiency of the cooling ability of different morphological exploitations to façade panels. The research process diagram in Figure 1 illustrates this and identifies the way in which each iteration informs the next.

### Experiment Set-Up

**Figure 2:**
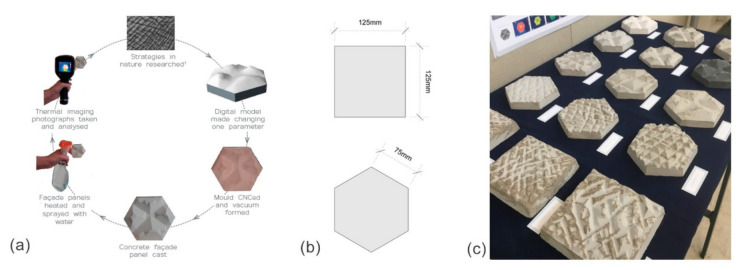
(**a**) Experiment process—studying morphology from nature, constructing a digital model, creating the physical model using CNC, forming a mold using vacuum forming around the physical model, casting a concrete panel in the mold, heating panels, spraying with water, taking thermal images over 30 min; (**b**) sample size—all of the sample panels have the same perimeter dimensions, dependent on their shape; (**c**) Photograph of the resultant textured panels for the various investigations.

**Figure 3:**
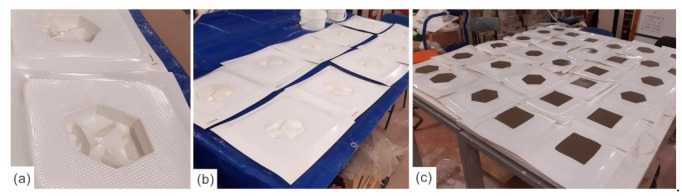
Photographs illustrating the casting process. (**a**) Vacuum formed mold from negative CNC’ed model (**b**) Vacuum form molds (**c**) Concrete casting.

The experiments were designed to examine the cooling behavior of concrete panels for different configurations of surface textures initially inspired by elephant skin. They aim to investigate the heat loss capabilities in a comparative way, where a set of investigations focus on the impact of defined variables related to wrinkles of skin, such as thickness, assembly, depth, and size on temperature drop. The sample façade panels have been realized by digitally modeling the texture of an elephant’s skin, from a detailed photograph, using SketchUp (which allowed the surface areas and volumes to be measured). A mold has then been CNC’ed and vacuum formed before being cast in concrete, as demonstrated in Figure 2 and Figure 3. The panels all have the same perimeter dimensions (Figure 2) and have the same concrete mix, unless otherwise stated. The mix comprised of two parts sand to one part cement. The sand comprised of two different grades, one part Holm sand (1–4 mm) and 2 parts limestone grit (0–6 mm). Each investigation had its own set of panels that were treated equally: heated for the same length of time (3 min) in the same oven (setting: 200 degrees Celsius, and 20 cm distance from the heating element), simultaneously. The panels were placed in room temperature (20 °C ± 1, ~40% RH), and then water (at room temperature) was sprayed three times onto the panels from a 30 cm distance. Thermal imaging photographs were then taken of each set of panels in 5 min intervals for 30 min. A FLIR E5 thermal imaging camera was used set to the Thermal MSX mode with rainbow color palette, matt emissivity and the temperature scale was locked at the beginning of each experiment (accuracy of ±2% of reading). The panels were tested simultaneously, ensuring they were subject to the same environmental conditions. This process was repeated three times with same procedures, and an average heat loss was calculated for each panel. The maximum temperatures of each panel at the start and end of the tests were recorded from the thermal imaging photographs. This identified the temperature drop for each panel over the duration of each test, providing insight into the impact of certain manipulations (the independent variables) and the way in which they informed the next iterations. Each set of investigation included controls in order to increase the reliability of the results through a comparison between control measurements and the other measurements. For the sake of brevity, only one set of thermal imaging from the three repeated tests is included in the paper as an example to illustrate the results, and the heat loss is provided as an average of the three tests.

## 4. Experiments and Results

### 4.1. Investigation 1: Proof of Concept

**Figure 4:**
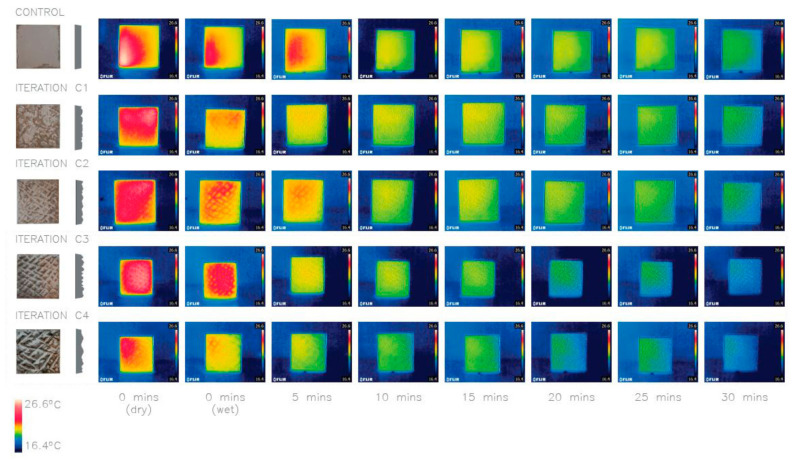
Thermal images of investigation 1: comparison of five concrete panels with different texture depths (d) and scale (s) and SA:V ratios. Control: plane (1.07 SA:V); C1: d–2.5 mm s–1 (1.16 SA:V); C2: d–5 mm s–1 (1.25 SA:V) C3: d–10 mm s–1 (1.33 SA:V); C4: d–10 mm s–2 (1.18 SA:V). Average heat loss after 30 min: Control 6.3 °C, C1 6.5 °C, C2 6.6 °C, C3 7.6 °C, C4 6.7 °C.

The first investigation (proof of concept) focused on the idea of introducing texture on to the surface to facilitate evaporative cooling. The thermal images in Figure 4 show that all textured panels (C1–4) lost more heat on average than the smooth control panel, and the more surface roughness of deeper and larger ridges, the larger the surface area to volume (SA:V) ratio becomes, and the more the heat loss occurs. These results lay the foundation for the study by investigating, in a comparative way, the impact of introducing surface texture and different roughness for evaporative cooling.

### 4.2. Investigation 2: Assembly of Morphology

The network of crevices across an elephant’s skin enhances thermal regulation by aiding water retention and therefore evaporative cooling. The morphological adaptation of their skin appears at various scales from the folds of skin to fractures of the stratum corneum [19]. The organization of these fractures creates troughs around the skin papillae organized in a hexagonal arrangement. Additionally, the honeycomb conjecture, proved by Hales [24], explains that hexagons have the largest ratio of area to perimeter, making them the most efficient tessellating polygon. The main aim of this investigation is to examine the impact of assembly of morphology on heat loss.

**Figure 5:**
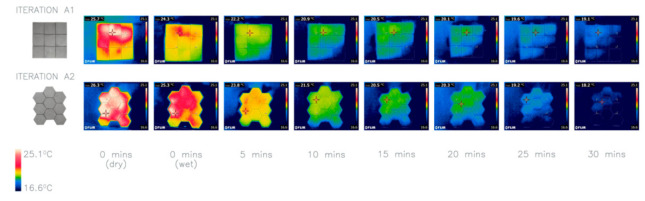
Thermal images of investigation 2: comparison of two different shapes with smooth surfaces and same SA:V ratio (2.39). Average temperature drops by 6.6 °C for the square and 8.1 °C for the hexagon.

Iterations A1 (9 square panels) and A2 (9 hexagonal panels) explore how the shape of the panel can affect cooling by comparing how they work collectively. The panels were cast without a texture to focus the investigation on the assembly of two panel shapes. The results (Figure 5) clearly illustrate that the hexagonal panels cooled more than the square panels, with temperature drops of 8.1 °C and 6.6 °C, respectively. It is proposed that this is due to the hexagonal pattern maximizing the collective surface area. The hexagonal panels appear to be more efficient and effective in packing and cooling, hence have been adopted for the rest of the investigations.

### 4.3. Investigation 3: Texture Depth

Iterations D1 to D6 explore the depth of the texture; D1 has the shallowest crevices with a SA:V ratio of 1.27 and D6 has the deepest crevices with a SA:V ratio of 1.47. It was hypothesized that the deeper the texture and the larger the SA:V ratio, the larger the water retention and the more heat dissipated.

**Figure 6:**
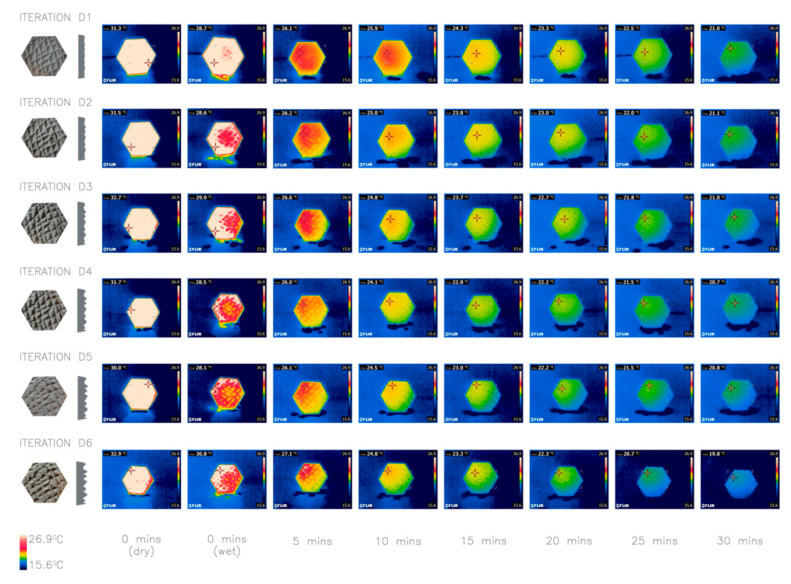
Thermal imaging of investigation 3: comparison of six iterations D1–D6 with different texture depths. Average heat loss after 30 min: D1 7.7 °C, D2 8.36 °C, D3 9.06C, D4 9.16 °C, D5 7.3 °C, D6 9.97 °C.

Additionally, using Bergmann’s rule [25], it could be theorized that the larger the SA:V ratio, the larger the capacity for the panels to lose heat regardless of the water retention. This is supported by Allen’s rule [26] which explains how peripheral parts of animals are larger where the temperature is higher, which increases their SA:V ratio to allow animals to stay cool. This can be witnessed in elephants as Weissenböck, Weiss, Schwammer and Kratochvil [17] illustrate that their ears have evolved to act as thermal windows by utilizing a network of subcutaneous vessels and their large surface area. The results of the investigation confirmed that the deeper the crevices the larger the SA:V and the drop in temperature (Figure 6). As a result, a deep texture was taken forward in the next iteration.

### 4.4. Investigation 4: Texture Scale

Texture scale was tested in parallel with the texture depth. Consequently, the panels in this investigation have a consistent depth of texture, and only the scale of the texture varies. The pattern of the texture is also the same, albeit less pattern the larger the scale. Iteration S1 had the smallest scale of texture with a SA:V ratio of 1.36 compared to iteration S6 with the largest scale of texture and a SA:V ratio of 1.14.

**Figure 7:**
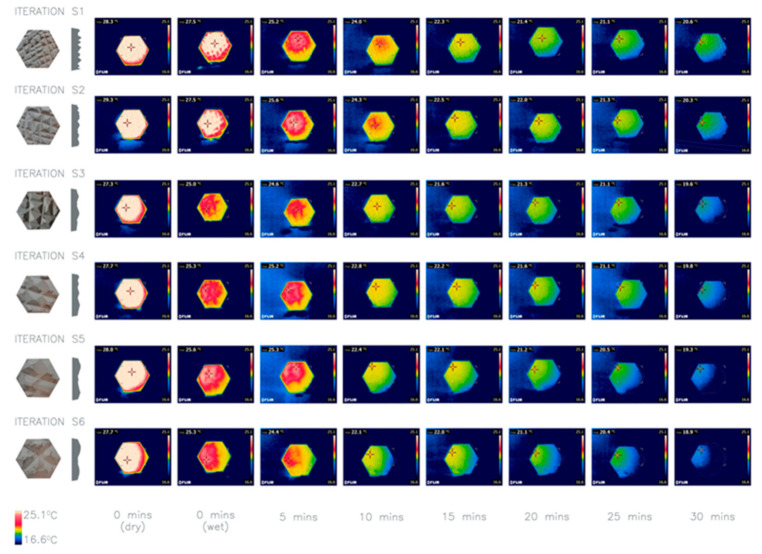
Thermal imaging of investigation 4: comparison of six iterations S1–6 with different texture scales. Surface area to volume ratios: S1 1.36, S2 1.24, S3 1.19, S4 1.17, S5 1.16, and S6 1.14. Average heat loss after 30 min: S1 8.96 °C, S2 9.8 °C, S3 7.66 °C, S4 9.13 °C, S5 8.8 °C, S6 9.43 °C.

The results of the average heat loss for the different iterations varied and the surface area to volume ratio was not consistent with the heat loss rate, as would be expected. By observing the thermal images (Figure 7), with similar starting temperature, iteration S6 appears to be coolest at the end of the experiment. Considering this difference, iteration S6 has been combined with the depth of D6 creating a deep and large-scale texture that will be taken forward for further investigations. For future investigations the involved variables need refinement and simplification in order to understand the correlation between their combinations and heat loss rates, preferably in an outdoor environment.

### 4.5. Investigation 5: Panel Colour

Radhi et al. [27] research into the color of building surface materials indicates that white, or light colored materials performed better at reflecting heat and concluded that it is beneficial that this is adopted to reduce the impact of the overheating of surfaces. This can also be seen in nature, commonly in ectotherms such as bearded dragons where their skin changes color for thermoregulation. This investigation examines the impact of color variation on the thermal performance of panels. This is explored by comparing grey concrete in Co1 to iteration Co2 which has a pigment added to create a black concrete panel and iteration Co3 which uses snowcrete to create a white concrete panel.

**Figure 8:**
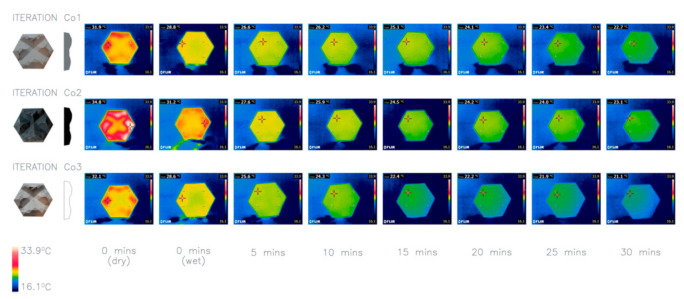
Thermal imaging of investigation 5: comparison of three iterations Co1–Co3 with different colors. Average heat loss after 30 min: Co1 9.1 °C, Co2 10.47 °C, Co3 8.8 °C.

Thermal test results show that the average heat loss for Co1 and Co2 was higher than for Co3, Figure 8. When viewing the thermal imaging it is clear that Co3 consistently reflected more heat than the others due to its color and therefore had a lower temperature at the beginning and end of the 30 min tests. This reinforces existing knowledge and shows that the larger the albedo of the panel the cooler the panel will be. It can, therefore, be concluded that the white concrete panels performed the best when the application to the built environment is considered, and Co3 has been taken forward to inform future iterations, despite the heat loss being the smallest.

Additionally, it has been shown that along with color, weathering can affect albedo and consequently the solar reflectance of concrete [28]. It is, therefore, deemed that further research is required into the concrete mix of the panels, if concrete were to be the selected material when adopting this product in the built environment. It is also noted that many organisms in warm environments have dark skin, and organisms in cold environments are often light in color. However, it is considered that organisms have not only evolved to climatic conditions for thermoregulation but also other environmental conditions such as for camouflage from predators [26].

### 4.6. Investigation 6: Material

For this study concrete has been used, as it is available and easily malleable allowing testing of various parameters and facilitating progression in this area of research. However, this does not imply that concrete is the most suitable material. This is highlighted by Radhi, Assem and Sharples [27] research into building surface materials which explores both color and heat storage capacity and its effect on urban heat islands. Regarding concrete, as it has a high thermal mass, its ability to store heat is large meaning it reaches a higher surface temperature than other materials with a lower thermal mass. This, therefore, contributes to the urban heat island effect and means that concrete may not be the most appropriate material for these façade panels. However, if concrete were to be adopted it may be reinforced with glass fibers for added strength to allow panels to be made larger and thinner. This is common in existing products.

**Figure 9:**
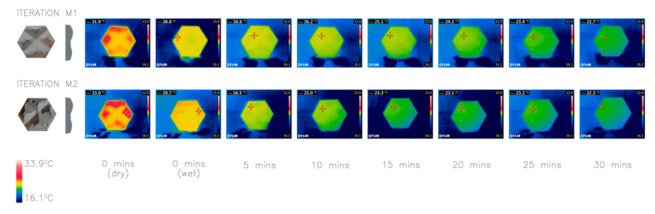
Thermal imaging of investigation 6: comparison of two iterations M1 and M2. Average heat loss after 30 min: M1 9.1 °C, M2 9.8 °C.

The main aim of this investigation was to measure the impact of adding glass fibers into the mix for added stability on the heat loss capabilities. The thermal imaging of M1 and M2 (Figure 9) shows that the difference was not significant therefore, we concluded, that added glass fibers would not adversely impact heat loss. The reinforcement would allow thinner tiles to be made, enlarging the SA:V ratio, which as Investigation 8 demonstrates, would help enhance the performance of the panels further. The use of glass fiber reinforcement, therefore, informs future iterations.

### 4.7. Investigation 7: Hydrophobicity

Darkling beetles utilize hydrophilic and hydrophobic qualities to collect and direct water [29]. This has been considered as a way to enhance the texture of the façade panels to direct water into the crevasses of the texture by making the tips hydrophobic. It is hypothesized that this would enable water to be retained for longer as water would move to the self-shaded areas of the panels.

**Figure 10:**
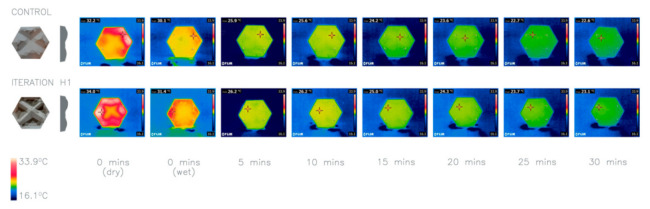
Thermal imaging of investigation 7: comparison between two iterations H1 compared to a control panel. Average heat loss after 30 min: Control 9.1 °C, H1 10.2 °C.

The results (Figure 10) illustrate that iteration H1 is cooler after 30 min. It should be noted that there is a limitation to the methodology as it does not test the shading properties of the façade panels, and therefore, it is unable to rigorously test the effects this may have on the performance of the panels. If this line of research is continued in future research, it is recommended that this should inform the methodology. Therefore, this has not been taken forward for future iterations. However, this needs further research to explore hydrophobicity implications further.

### 4.8. Investigation 8: Panel Thickness

**Figure 11:**
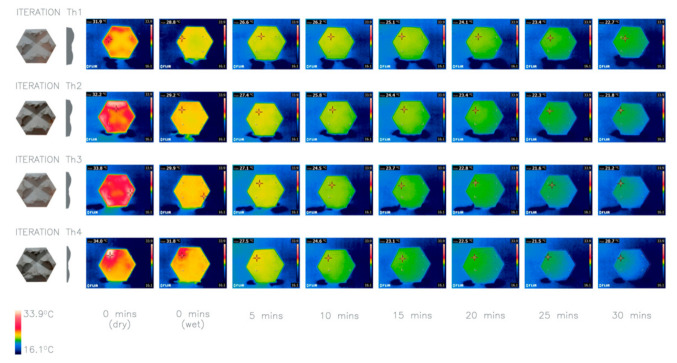
Thermal imaging of investigation 8: comparison of four iterations Th1–4 with different thicknesses. Average heat loss after 30 min: Th1 9.1 °C, Th2 11.73 °C, Th3 13.23 °C, Th4 14 °C.

The aim of this investigation is to examine the impact of different panel thicknesses on their thermal performance. Iteration Th1 is the thickest panel with the smallest SA:V ratio of 1.09 and Th4 the thinnest with the largest SA:V ratio of 1.74. The results are consistent with Bergmann’s rule [25], where the thinner the façade panel, the larger the SA:V ratio and, therefore, the larger the temperature drop of the panel. The iterations were heated simultaneously for the same length of time, with the results (Figure 11) showing that the thinner the panel the faster the panel heats up, and therefore it had a higher initial temperature. For this investigation, the impact of heating the panels for the same length of time rather than heating the panels to the same starting temperature has been evident. It is important to note that this decision was made to emulate the application of the panels within the built environment, as façades will be warmed for a set period of time during the day.

### 4.9. Investigation 9: Patterns

**Figure 12:**
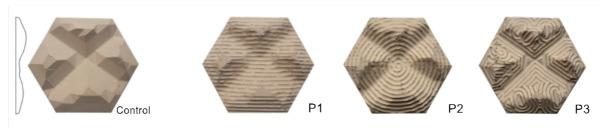
Iterations of patterns: P1—horizontal lines; P2—spiral lines; P3—curvy random lines.

**Figure 13:**
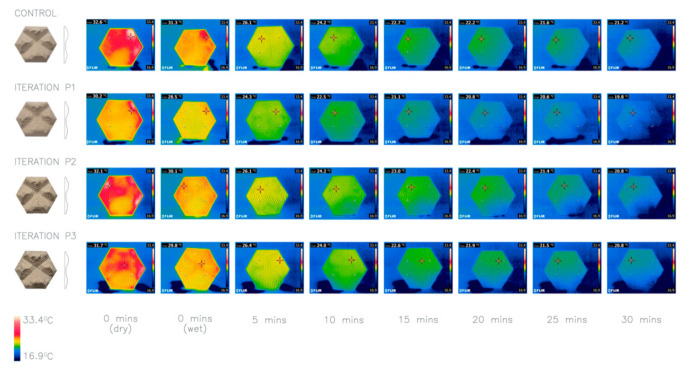
Thermal imaging of investigation 9: pattern introduction onto a textured panel. Iterations P1–3 compared with a control panel. Average heat loss after 30 min: Control 14.16 °C, P1 13 °C, P2 13.43 °C, P3 12.86 °C.

As aforementioned, maximizing the SA:V ratio, increases heat dissipation. Adding three different patterns of lines (Figure 12) has been undertaken to explore the cumulative effect of different patterns upon a texture. The patterns were created by the finishing pass of the CNC machine. A 4mm drill bit was used at 100% stepover to create lines of ridges in the three different configurations. The horizontal (P1), spiral (P2) and curvy (P3) surface patterns were tested against a control panel without a surface pattern. The thermal imaging (Figure 13) demonstrates that the average heat loss for the added pattern did not increase heat loss compared to the control, as would have been expected due to increased SA:V ratio. Therefore, further experimentation is needed to clarify the impact of this type of surface patterns on cooling in an outdoor environment. For the purposes of this study, we took P1 forward to visualize the potential application, but further research into pattern composition will be taken in future studies.

## 5. Discussion and Conclusions

This paper examines the impact of some morphological (inspired by nature) and material variables on the thermal performance of concrete panels by using thermal imaging. Overall, the outcome of this research has identified that textured façade panels can aid cooling, which could have a significant impact on the built environment and opens new avenues and areas to explore in ongoing and future studies related to thermoregulation.

**Figure 14:**
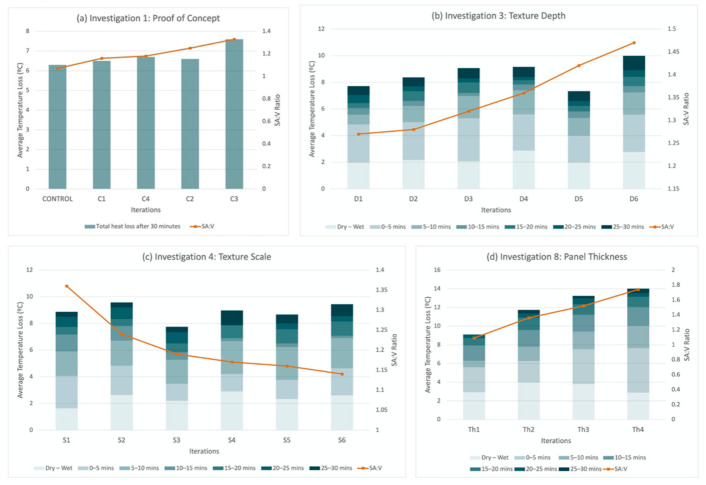
Graphical analysis illustrating the correlation between the SA:V ratio and heat dissipation for different investigations at 5 min intervals: (**a**) Investigation 1—Proof of Concept; (**b**) Investigation 3—Texture Depth; (**c**) Investigation 4—Texture Scale; (**d**) Investigation 8—Panel Thickness.

The results show that the general assembly of hexagonal panels is not only effective in packing but can also facilitate cooling. In general, deeper textures have a larger heat loss capability. Thinner panels, and white panels help to ensure the temperature of the panels remain cool. The glass fiber reinforcement can allow the panels to be thinner without affecting their heat loss capabilities. Furthermore, the impact of different SA:V ratios on heat loss capabilities vary for different surface roughness, i.e., smooth and textured. For panels with the same texture, but varying depths of texture or thickness and consequently varying SA:V ratios, the SA:V ratio was consistent with heat loss capabilities (Figure 14a,b,d). For panels with varying scales of texture, the heat loss behavior did not follow a clear trend with SA:V ratios (Figure 14c). Investigation 9 opened some questions related to adding a refined pattern on a textured panel, which has some relevance to the work of Grobman and Elimelech [2]. Reflecting on these, the correlation between surface morphology, SA:V, and heat loss capabilities needs further investigation in order to better understand the impact of the microclimate that these different texture scales create due to convection on evaporative cooling. Further experimentation regarding different combinations of scale, hydrophobicity and surface texture would help to establish a better understanding on the correlation with heat loss capabilities. The material of the panels can also vary by exploring different materials with different conductivities and absorption properties, which might affect water retention, heat capacity, and evaporative cooling. The effects of weathering on these materials and the subsequent impact on their albedo can also be explored.

**Figure 15:**
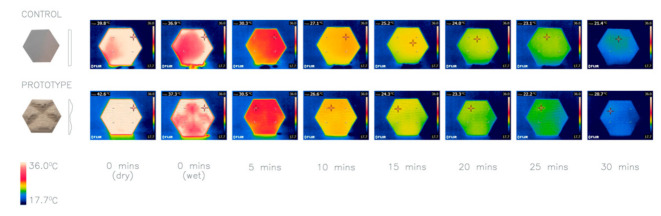
Thermal imaging of a prototype with P1 compared with a plane panel. Average heat loss after 30 min: Control 20.23 °C, Prototype 21.9 °C.

**Figure 16:**
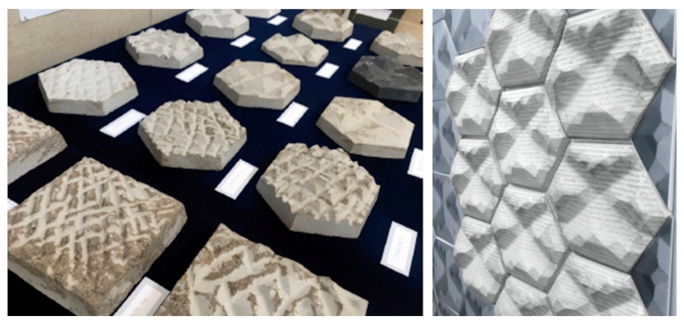
Left: Photograph of the cumulative textures of the various investigations, and right: The assembly of a potential application using P1 prototype.

A potential application of iteration P1 (as an example) is demonstrated in Figure 15 and Figure 16. The thermal imaging comparing it to a plane concrete panel of the same perimeter dimensions and weight allows for reflection on the overall performance of the panel (Figure 15). These indicate that the use of textured façade panels could improve the thermoregulation of buildings and potentially reduce its energy demands by reducing the need for mechanical cooling.

In summary, this paper argues that the exploitation of texture to façades can be used to aid heat loss through evaporative cooling. This could help to tackle the overheating of buildings, through a simple and achievable solution in warm temperature environments and opens new opportunities to solving overheating problems by complementary morphological strategies for passive cooling inspired by nature. We hope to expand this morphological design investigation, aiming to develop a framework for designers that brings in applicable passive architectural thermal solutions for existing and new buildings.

## Figures and Tables

**Table 1:** Examples of evaporative cooling found in nature.

Example	Adaptation Means	Mechanism
Humans	Physiological	Sweat [12]
Elephants	Morphological	Wrinkly skin [1]
Kangaroo	Behavioral	Saliva spreading on forearms [14]
Vultures	Behavioral	Urohidrosis [15]

## Data Availability

All data are available within the manuscript.
